# RYGB increases postprandial gastric nesfatin-1 and rapid relieves NAFLD via gastric nerve detachment

**DOI:** 10.1371/journal.pone.0243640

**Published:** 2020-12-10

**Authors:** Geng Wang, Qingbo Wang, Jie Bai, Gang Li, Kaixiong Tao, Guobin Wang, Zefeng Xia

**Affiliations:** 1 Department of Gastrointestinal Surgery, Union Hospital, Tongji Medical College, Huazhong University of Science and Technology, Wuhan, China; 2 Department of Gastrointestinal Surgery II, Renmin Hospital of Wuhan University, Wuhan, China; Medical University Innsbruck, AUSTRIA

## Abstract

**Background:**

Roux-en-Y gastric bypass (RYGB) could reduce nonalcoholic fatty liver disease (NAFLD) ahead of the weight-loss effects. But the detailed mechanisms remain unclear.

**Material and methods:**

A high-fat diet (HFD) was fed to induce obesity. RYGB was then performed. Gastric nesfatin-1 was measured by enzyme-linked immunosorbent assay (ELISA) in portal vein and polymerase chain reaction (PCR) in gastric tissues. Modified surgeries including vagus-preserved bypass and vagectomy were performed and postprandial gastric nesfatin-1 were analyzed. The effects of nesfatin-1 on hepatocytes were studied by PCR and immunohistochemistry. Both intraperitoneal and intracerebroventricular injection (ICV) were performed to analyze the *in vivo* effects on liver lipid metabolism.

**Results:**

Increased postprandial portal vein nesfatin-1 was observed in RYGB but not in control groups. This increase is mainly due to induction of gastric nesfatin-1. A modified RYGB in which the gastric vagus is preserved is conducted and, in this case, this nesfatin-1 induction effect is diminished. Mere vagectomy could also induce a similar nesfatin-1 increase pattern. The infusion of nesfatin-1 in the brain could inhibit the expression of gastric nesfatin-1, and the effects are diminished after gastric vagectomy. *In vivo* and *in vitro* nesfatin-1 stimulation in the liver resulted in improvements in lipid metabolism.

**Conclusions:**

Severing the gastric vagus during RYGB could cut off the negative control from the central nervous system (CNS) and result in increased postprandial gastric nesfatin-1 post surgery, which in turn, improves NAFLD.

## Introduction

Obesity has become an enormous public health problem [1,2,6]. Obesity further leads to hypertension, type 2 diabetes mellitus (T2DM), and non-alcoholic fatty liver disease (NAFLD) [3–6]. Bariatric surgery is confirmed to be a very efficient and consistent therapy [7,8]. Roux-en-Y gastric bypass (RYGB) is one of the commonly performed surgeries. RYGB not only confers excellent early and sustained weight loss, but also resolves weight-related comorbid conditions, including NAFLD [9–11].

Among several modalities for NAFLD, RYGB is considered to achieve rapid, significant, and sustained improvement [9,12]. Interestingly, previous reports suggested that the anti-NAFLD effects are not due to weight loss alone. Unlike other pure volume-restrictive surgeries such as sleeve gastrectomy (SG), in RYGB there are changes in acid exposure to food [13]. In addition, gastric emptying of liquids and solids is markedly accelerated. Thus, gut microbiota and gut hormones are significantly affected by RYGB [13,14]. These changes could also affect the functioning of the liver.

In 2006, Oh-I and colleagues were the first to report the function and secretion of nucleobindin 2 (NUCB2)/nesfatin-1 [15]. NUCB2/nesfatin-1 is expressed in the neuronal soma and primary dendrites. It is also very abundant in the gastric mucosa [15,16]. Previous reports showed that nesfatin-1 could control appetite, reduce food intake, inhibit gastric emptying, and affect the metabolic conditions of adipose tissue, islet, muscle, and liver [15,17,18]. In particular, NUCB2/nesfatin-1 is involved in glucose and lipid metabolism [18,19]. Clinical reports showed that patients with NAFLD had lower levels of serum nesfatin-1 [20]. Interestingly, studies using rodent models have suggested that peripheral infusion of nesfatin-1 could decrease hepatic steatosis through the adenosine monophosphate (AMP)-activated protein kinase pathway [21]. However, evidence of how nesfatin-1 affects liver metabolism is still lacking.

In light of the findings on nesfatin-1 and RYGB, we tried to study the regulation of peripheral nesfatin-1 by surgery. By performing slight changes in the surgery model, we attempted to identify the key bridge for the crosstalk between the central nervous system (CNS) and peripheral tissues in the regulation of nesfatin-1. Therefore, this study aims to confirm a mechanism that is independent of weight loss. The findings of the study show a special effect of surgery on NAFLD that, could not be achieved with conservative treatment.

## Materials and methods

### Animals and diets

All animal experiments complied with the guidelines of the Huazhong University of Science and Technology animal management program. 7-week-old Sprague Dawley (SD) rats with similar body weights (180–200 g), purchased from the Animal Breeding Center of Tongji Medical College, Huazhong University of Science and Technology. The rats were housed in a specific pathogen-free (SPF) animal room (24°C, 12-hour-light/dark cycle with lights on at 7:00 am) and were induced to become obese for 10 weeks with a high-fat diet (HFD, D12492, 60% high fat, BEIJING HFK BIOSCIENCE). The body weights of the rats ranged from 725 to 747 g before surgery. The rats were sacrificed with cervical dislocation before collecting samples.

### Perioperative nursing, anesthesia, and postsurgical recovery for all groups

For all groups undergoing different surgeries, the perioperative treatments were the same. At least 16 hours of fasting were required before surgery. Oral rehydration salts were permitted during fasting. The rats were anesthetized with pentobarbital sodium [35–40 mg/kg intraperitoneally (IP)] [22,23]. After the anesthesia was successful, the rat was placed on an operating table kept at a constant temperature (set at 37°C). The abdominal skin was disinfected with 1% polyninylpyrrolidone, and a midline incision (1.5–2 cm under the mucronate cartilage) was made to enter the abdominal cavity. After the required surgical, 10 mL of 5% glucose and sodium chloride were injected subcutaneously [24].

### Postoperative nursing

Therapies were based on previous reports. The rats were placed on a 37°C constant-temperature heating pad and rewarmed for 12 hours. Food was supplied only 24 hours after surgery. During this time period, 10 mL of 5% glucose and sodium chloride were administered subcutaneously [25] three times a day. Oral 5% glucose solution was given 2 to 4 days after surgery. Boiled eggs were given 5 to 7 days after surgery [26,27]. A preoperative diet was resumed 1 week after surgery.

### Introduction of different surgery procedures

#### RYGB (an anatomical diagram is shown in S1A Fig)

The abdominal cavity was entered, and the ligament of Treitz was identified, where the proximal jejunum fixes to the ileum. The jejunum was separated and severed approximately 10 cm below the Treitz ligament, and the two broken ends were completely and firmly closed by suture to avoid leakage after surgery (SILK 4–0 Ethicon) [24,26]. The ends were named as the proximal jejunum (biliopancreatic limb) and distal jejunum (alimentary limb) according to their location. The ileocecal junction site was identified, and the proximal jejuno-ileostomy was completed 25 cm from the site by performing side-to-side anastomosis using continuous sutures (PDS 6–0 Ethicon) [28,29]. The tissue around the stomach was cleaned, and the gastrohepatic and gastrosplenic ligaments were excised to facilitate placing the stomach outside the abdominal cavity. A 5-mm incision was opened in the greater curvature of the stomach, where there is a low density of vascular networks, and the inside of the stomach was cleaned. The incision was then sutured and a stomach pouch was constructed. An anastomosis was performed between the stomach pouch and the distal jejunum (PDS 6–0 Ethicon). The abdominal cavity was washed with 37°C-40°C warm saline, and a double check for potential hemorrhage and intestine arrangements was performed before the abdominal wall was closed.

#### Sham surgery

For the sham surgery group, operators closed the abdominal cavity after opening the abdominal cavity and freeing the stomach.

#### Vagus preserved bypass (modified RYGB) (An anatomical diagram is shown in S1B Fig)

In this group, we did not establish a gastric pouch and instead directly anastomosed the distal end with the stomach. The gastroesophageal region was unaffected, and thus the vagus was preserved. The remaining procedure was the same as that for the standard version of RYGB.

#### Vagectomy

In the vagectomy group, the perigastric ligaments and mesentery were dissociated, and the stomach was drawn to the abdominal surface for the convenience of locating the anterior and posterior branches of the gastric vagus nerve **(S1C and S1D Fig)** and was then excised with ophthalmic scissors. Operators took care to avoid damage to the surrounding blood vessels. After the operation, the abdominal cavity was cleaned with 37°C sterile saline, and the anastomosis and abdominal cavity were carefully checked. The other steps were the same as those in the RYGB group.

#### Performance of different procedures

The names and descriptions of all groups and subgroups are listed in **Table 1 and S2–S4 Figs**.

**Table 1:** Names and introductions of the groups and subgroups.

**Fig 1**	
**RYGB**	Rats which received RYGB surgery
**Sham surgery**	Rats which received open and close surgery
**Control**	Rats which did not receive surgery
**Fig 2**	
**Modified RYGB**	Rats which received RYGB with stomach vagus preserved as shown in Fig 2A images
**Vagectomy**	Rats which received vagectomy
**Fig 4**	
**Control**	Treatment of saline for *in vitro* or *in vivo*
**Nesfatin-1**	Treatment of nesfatin-1 for *in vitro* or *in vivo*
**Non-treatment**	No special treatment for *in vitro* or *in vivo*

### Blood collection

Rats were housed in metabolic cages 12 hours before blood collection for fasting. The rats were anesthetized, and in the fasting state, blood was collected at the 0-hour time point. An oral gavage of 50% glucose (1 g/kg, body weight) was administered [30]. Blood samples from the medial canthus vein (peripheral vein) and portal vein were collected at 0.5, 1, 1.5, 2, 2.5, and 3 hours after oral gavage. Heat-preservation facilities were provided during the collection process.

The portal vein was surgically exposed under anesthesia for blood collection. After insertion of a 23-gauge needle into the vessel, a catheter was inserted into the portal vein of the rat. The needle was then slightly lifted ventrally to act as a guide for the polyethylene tubing used for catheterization. When the needle was removed, the catheter was fixed into place by a drop of cyano-acrylate ester cement. For each time point, 50 μL of blood was collected and centrifuged for the collection of plasma [31].

### Iodine water radiography

After the rats were anesthetized, 2 mL of iohexol was administrated via oral gavage. The rats were placed into the small animal *in vivo* imager (*In-Vivo* FX PRO, Bruker) forradiographic examination. Images were taken every 10 minutes over a 60-minute period. The images were then analyzed and compared.

### Rodent sample collection

To study nesfatin-1 expression, 10 control rats and 10 RYGB rats were used and analyzed. In the control group, five rats were euthanized, and the stomach and duodenum were isolated and collected. The other five rats received an oral gavage of 3 mL 50% glucose [30]. Two hours after oral gavage, the stomach and duodenum were isolated and collected. The same treatment was performed on the RYGB rats (who received RYGB after 4 weeks). The stomach and duodenum were immediately divided into two parts. Approximately 50 mg of tissues was stored at –80°C. After all collections were completed, the samples were thawed for western blot assay. Approximately 50 mg of tissue were directly placed into Trizol and prepared for polymerase chain reaction (PCR) analysis [32]. For *in vivo* liver investigation, five control rats, five sham surgery rats, and five vagectomy rats were used for the analysis. The rats received an oral gavage of 3 mL 50% glucose after 2 hours. The rats were then euthanized, and the liver was obtained for hematoxylin and eosin (HE) staining and PCR analysis [33].

### Enzyme-linked immunosorbent assay (ELISA)

Blood samples were centrifuged, and plasma was collected. The supernatant was stored at –80°C. After all collections were completed, the samples were thawed for assay. Repeated thawing and freezing were avoided. Concentrations of nesfatin-1 in the serum at different time points were detected by the nesfatin-1 ELISA kit (Phoenix Pharmaceuticals Inc, California, USA. Catalog Number: EK-003-22. Intra-assay coefficients of variation <10%, Inter-assay coefficients of variation <15%. Sensitivity: ≤1.26 ng/ml.).

### IP injection and intracerebroventricular injection (ICV)

For IP and ICV experiments, 15 rats were divided into the nesfatin-1 group, nontreatment group, and control group. For the IP experiment, each rat in the nesfatin-1 group received 20 μg/kg nesfatin-1 through IP [34]. Stroke-physiological saline solution was administered to the control group, and no other treatment was given to the nontreatment and control groups. Gastric tissues and blood from the portal vein were collected for PCR and ELISA tests. For ICV experiments, similar therapies were performed through the ICV pathway. Details of the ICV technique were obtained from previous reports [35].

For further study of the ICV effects, 4 weeks after vagectomy, five rats in the vagectomy group, five control rats, and five fake surgery rats with similar body weight were randomly divided into the injection (ICV) group, control group, and nontreatment group. The rats in the injection and control groups were anesthetized with pentobarbital sodium and fixed on the brain stereotactic locator. The hair was removed from the head of the rat, and the skin was sterilized with iodophor. A slit of approximately 1 cm in length was cut along the sagittal line with a scalpel at the midpoint of the two ears of the rat, and the subcutaneous tissue was bluntly separated by ophthalmic tweezers to completely expose the fonticulus anterior. The cross-point of the fonticulus anterior was set as the zero point, and the lateral ventricle was located on the surface of the skull with reference to the rat brain stereotaxic map (George Paxinos and Charles Watson). The positioning coordinates werer AP: 1.0 mm, L: 1.5 mm, H: 4.5 mm. A skull drill was used to drill a hole of approximately 1 mm in diameter on the surface of the rat skull (to prevent excess bleeding, drilling was stopped when there was a sense of breakthrough). Thereafter, nesfatin-1 (25 pmol) was extracted with a micro syringe and injected into the paracele through a positioning point. After injection, the micro syringe was kept in situ for 5 minutes and then pulled out of the brain. The skin was sewn after the hole was sealed. Stroke-physiological saline solution (335 pmol) was administered to the control group, and no treatment was provided to the nontreatment group. Gastric and liver tissues were collected 2 hours after ICV for PCR, and portal vein blood of control rats ([ICV] group and control group) was collected at 0, 1, 2, and 3hours after ICV for ELISA.

### Cell cultures

Human normal liver cells (LO2) (80,000 units per hole) were implanted in the 12-hole plate. Twelve holes were randomly divided into two groups (NES-/NES+). Each group contained five samples. The nesfatin-1 group received nesfatin-1 (10^-8^M) stimulation for 24 hours [36]. The cells were then collected and tested.

### Real Time PCR (RT-PCR)

The total RNA was isolated by Trizol (9109, Takara, Otsu, Shiga, Japan), and subjected to RT-PCR, and then synthesized to cDNA using the Takara PrimeScript RT Master Mix (Takara, RR036A). The primer sequences are listed in **Table 2**. RT-PCR analysis was performed using an SYBR Green PCR Master Mix (Takara, RR420A) with the StepOne Plus Real-Time PCR System (Applied Biosystems, Grand Island, NY). The expression of acaca, dgat1, fasn, and FXR in the liver of different groups and the expression of nesfatin-1 in the stomach of different groups were detected.

**Table 2:** Details of the primers.

**gadph**	
forward	5’-TCAAGAAGGTGGTGAAGCAG-3’
reverse	5’-AGGTGGAAGAATGGGAGTTG-3’
**acaca**	
forward	5’-CTTGGGGTGATGCTCCCATT-3’
reverse	5’-GCTGGGCTTAAACCCCTCAT-3’
**dgat1**	
forward	5’-GGAGACCGCGAGTTCTACAG-3’
reverse	5’-CCTGGCCATCCATTTGTTGC-3’
**fasn**	
forward	5’-TGGAGCTTGTGT AGCCTTCG-3’
reverse	5’-TGGGACAGGTTGTAATCGGC -3’
**nesfatin-1**	
forward	5’-CAGTTTGAACACCTGAACCACCA-3’
reverse	5’-TCATGCCGAGTCCGGTCATA-3’

### Western blot assay

Related tissues were harvested and homogenized in lysis buffer. Proteins were subjected to sodium dodecyl sulfate–polyacrylamide gel electrophoresis with a 10% running gel and then transferred to a polyvinylidene difluoride membrane. Membranes were incubated for 1 hour at room temperature with 5% fat-free milk in Tris-buffered saline containing Tween 20, followed by incubation overnight at 4°C with primary antibody (nesfatin-1, R&D, the catalog number is AF6895, 2 μg/Ml). (Secondary antibody, R&D, the catalog number is HAF016, 1:1000 dilution). Specific reactions were detected using IRDye-conjugated secondary antibody and visualized using the Odyssey infrared imaging system (LI-COR Biosciences, Lincoln, NE).

### HE staining

The liver samples of each group were collected. And liver specimens were fixed with 4% paraformaldehyde and stained with HE [37].

### Oil red staining

For oil red staining experiments, oleic acid (62.5 μM) was added to all wells. The cells were then cultured for 24 hours. Subsequently, nesfatin-1 (10^−8^ M) was added to the NES+ group, whereas the NES–group was left untreated, and all cells were further cultured for 12 hours. The oil red O staining and hematoxylin staining were subsequently performed. Oil red staining protocols were based on previous reports [21].

### Statistical analysis

The data are shown as mean ± standard error of the mean. The statistical significance was evaluated by the Student t-test (2 groups) or one way ANOVA (3 groups). *p* < 0.05 was accepted as statistically significant. All analyses were performed using SPSS v 17.0 software (SPSS Inc., Chicago, IL).

## Results

### Postprandial nesfatin-1 in the portal vein is rapidly induced in rats after RYGB

**Fig 1:**
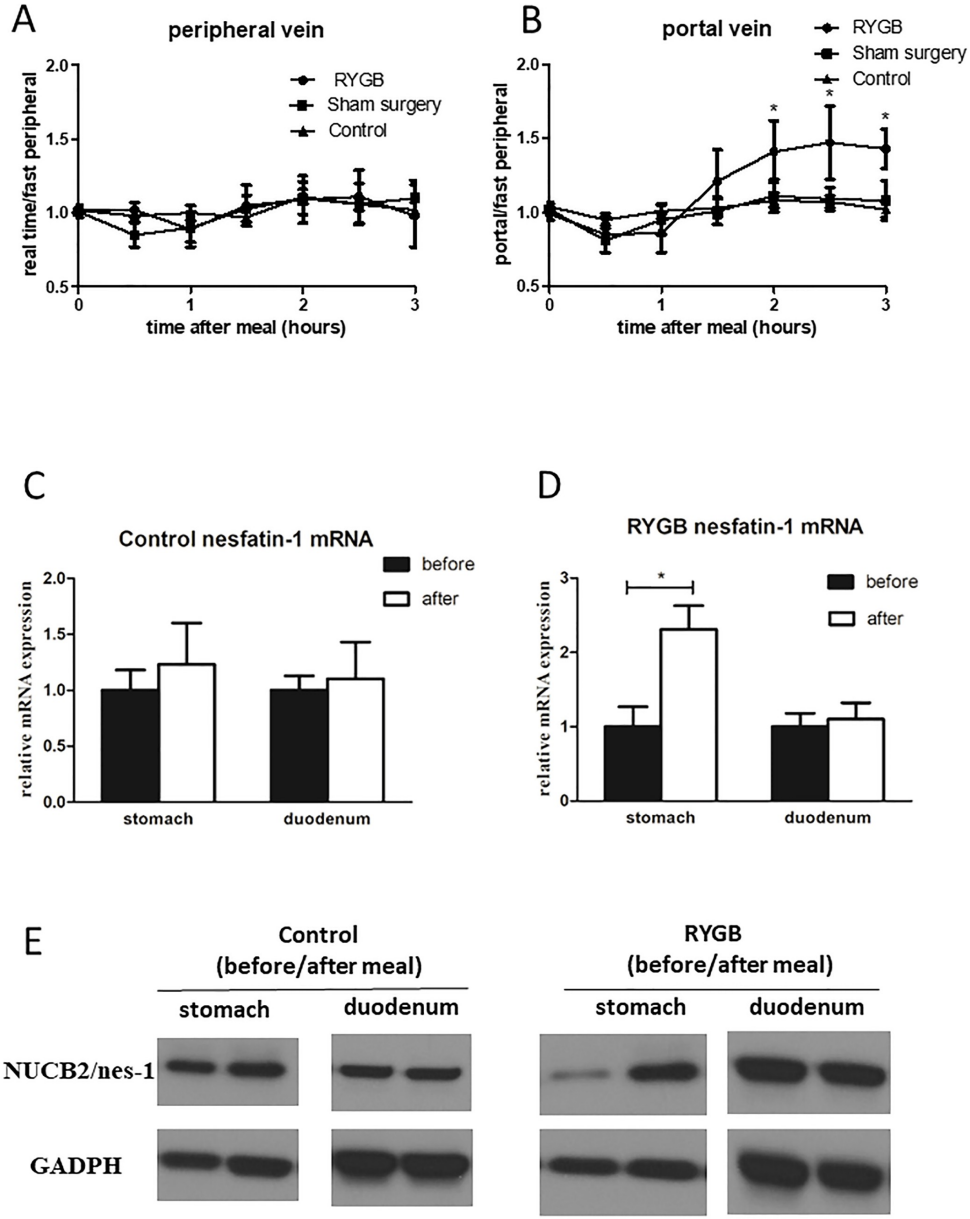
Overexpression of postprandial gastric nesfatin-1 after RYGB. A. Changes of nesfatin-1 concentration in peripheral blood of rats undergoing RYGB before and after meal. (n = 10 in each group, one-way ANOVA, p>0.05 in all time points). B. Changes of nesfatin-1 concentration in portal vein blood of rats undergoing RYGB before and after meal. Nesfatin-1 in portal vein blood began to rise 1h after meal and maintained until 3h after meal. (n = 10 in each group. one way ANOVA, 1h time point p = 0.003, 1.5h time point p = 0.003, 2, 2.5, 3h time point p<0.001). C. The expression of nesfatin-1 mRNA in gastric and duodenal tissues was compared with that in normal rats on a fasting state (before) and 2h after feeding (after). Relative to “before” and normalized to GADPH. The stomach and duodenum samples were calculated and compared separately. (n = 6 in each group, Student t test, p = 0.05 in both groups). D. The expression of nesfatin-1 mRNA in gastric and duodenal tissues was compared with that in rats with RYGB on a fasting state (before) and 2h after feeding (after). Relative to “before” and normalized to GADPH. The stomach and duodenum samples were calculated and compared separately. (*p<0.001 in stomach from RYGB rats, fasting state compare to 2h after feeding, n = 6 in each group, Student t test). E. The expression of nesfatin-1 protein in stomach and duodenum in normal rats and rats undergoing RYGB.

Previous studies indicated that nesfatin-1 is closely related to control of food intake. However, our findings showed no significant variations in plasma nesfatin-1 level before and after meals **(Fig 1A, as shown in control group and sham surgery group)**. A similar pattern was observed after RYGB **(Fig 1A, as shown in RYGB group)**. Because the portal vein collects peripheral blood from the gastro-intestine, we tried to assess the expression of nesfatin-1 of gastrointestinal origin. Interestingly, there were different regulation patterns among the control groups, sham surgery groups and the RYGB groups **(Fig 1A)**. Only in RYGB rats, there was a consistent increase in nesfatin-1 levels after meals **(Fig 1B, RYGB group)**. This finding indicated that the regulation pattern of gut nesfatin-1 is largely changed by RYGB. Because both the stomach and duodenum could secrete nesfatin-1, we further validated the expression in the two organs by PCR and Western blot, and the results suggested that the increase in nesfatin-1 is mainly due to induction in the stomach **(Fig 1C–1E)**.

### Increase in portal vein nesfatin-1 after RYGB is related to the severed gastric vagus nerve during surgery

**Fig 2:**
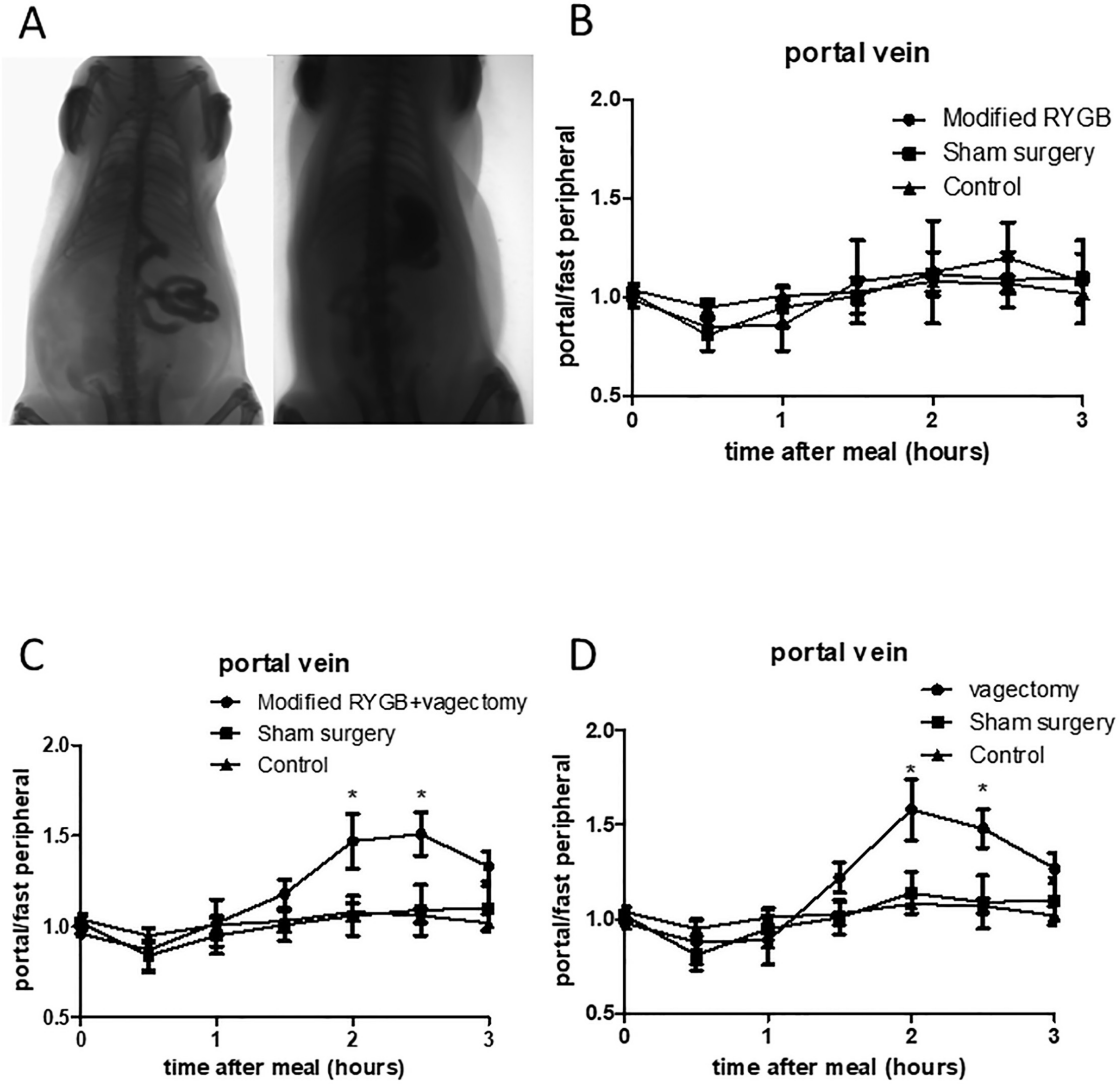
Roles of the gastric vagus during RYGB. A. Schematic diagram of iodine hydrography of RYGB with stomach preserved. Picture shows iodine water passing through the stomach into the duodenum. Classic RYGB was shown in the left while the vagus preserved RYGB (modified RYGB) was shown in the right. B. Changes of nesfatin-1 concentration in portal vein blood before (detecting nesfatin-1 concentration in peripheral vein blood) and after meal in rats undergoing RYGB with vagus preserved (modified RYGB) (n = 10 in each group, p>0.05 in all time points, one way ANOVA). C. Changes of nesfatin-1 concentration in portal vein blood before and after meal of rats undergoing modified RYGB plus vagectomy. (*p = 0.021 for 0.5h, *p<0.026 for 1h after meal, p<0.001 in 2, 2.5, 3h time point, n = 10 in each group, one way ANOVA). D. Changes of nesfatin-1 concentration in portal vein blood before and after meal in rats undergoing vagectomy. (*p<0.023 for 0.5h, *p = 0.02 for 1.5h, *p<0.001 for 2, 2.5 and 3h after meal, n = 10 in each group, one way ANOVA).

A modified RYGB surgery was performed as shown in **Fig 2A**. Interestingly, in the modified surgery, the peak of postprandial nesfatin-1 disappeared **(Fig 2B, modified RYGB group)**. In RYGB, the gastric vagus is cut off, whereas in modified RYGB, it is preserved. Thus, the vagus nerve may be the key point for the changed regulation pattern of nesfatin-1. To further verify this hypothesis, a modified surgery plus vagectomy was performed, and we again observed an increase in postprandial portal vein nesfatin-1. Furthermore, a simple vagectomy was performed without any bypass treatment. Interestingly vagectomy alone could also achieve a similar postprandial increase in nesfatin-1 **(Fig 2D, vagectomy group)**.

### Vagectomy could promote the degree of NAFLD via induction of postprandial gastric nesfatin-1

**Fig 3:**
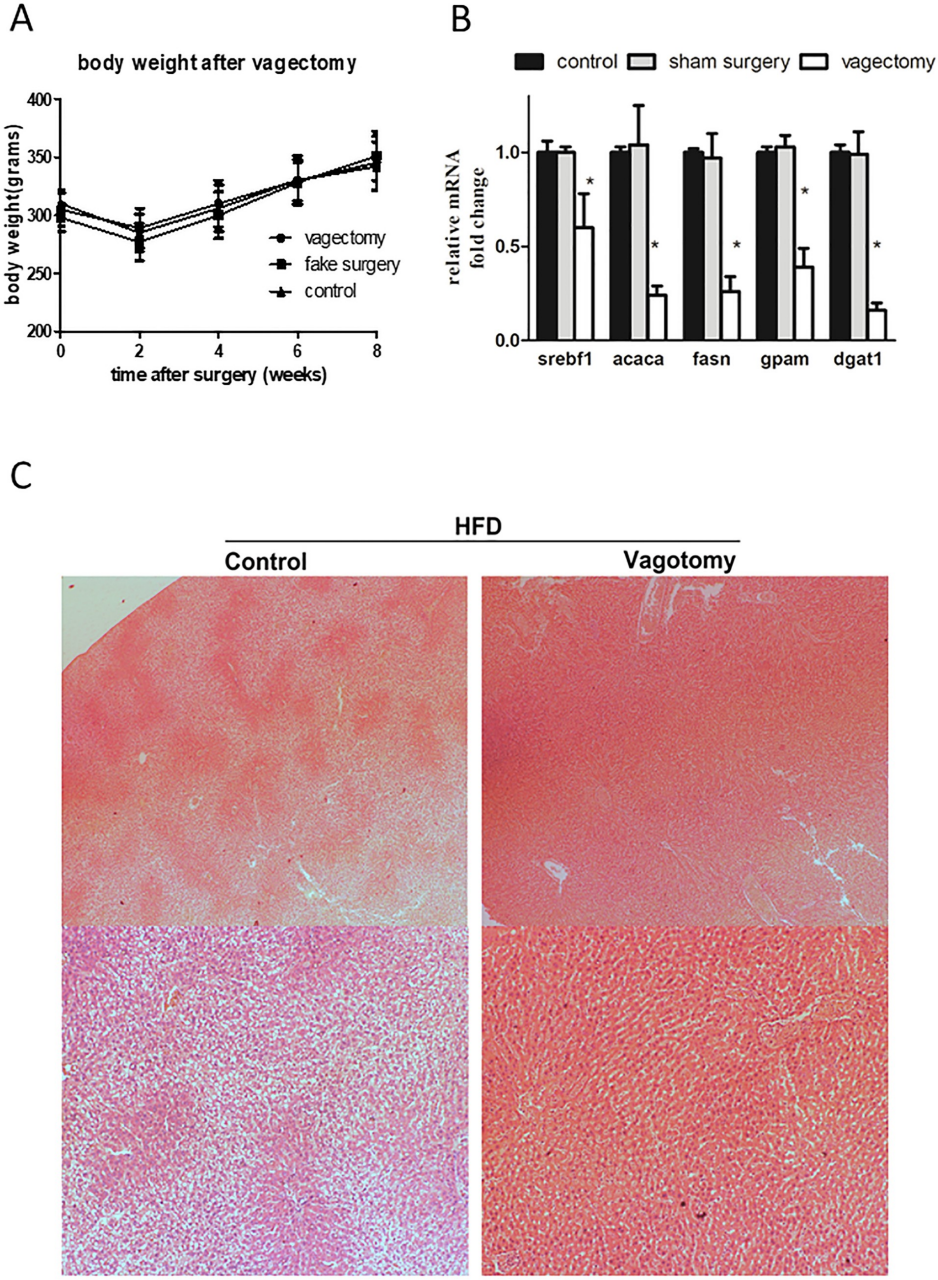
Effects of vagectomy on lipid metabolism. A. Analysis of body weight after vagectomy (repeated ANOVA, p = 0.987 for differences among 3 groups. p<0.001 for the “time after surgery” factor). B. Comparative analysis of liver lipid metabolism after vagectomy. For each target gene, relative to control and normalized to GADPH. (one way ANOVA, p<0.001 in all subjects). C. Comparison of liver sections between the control group and the vagectomy group.

We found that vagectomy alone does not change the body weight of rats **(Fig 3A)**. But, vagectomy could result in rapid improvements in terms of hepatic lipo-genesis and steatosis **(Fig 3B)**. Relief of NAFLD could also be observed by HE staining of liver sections **(Fig 3C)**.

### Nesfatin-1 improved NAFLD and lipogenesis *in vivo* and *in vitro*

**Fig 4:**
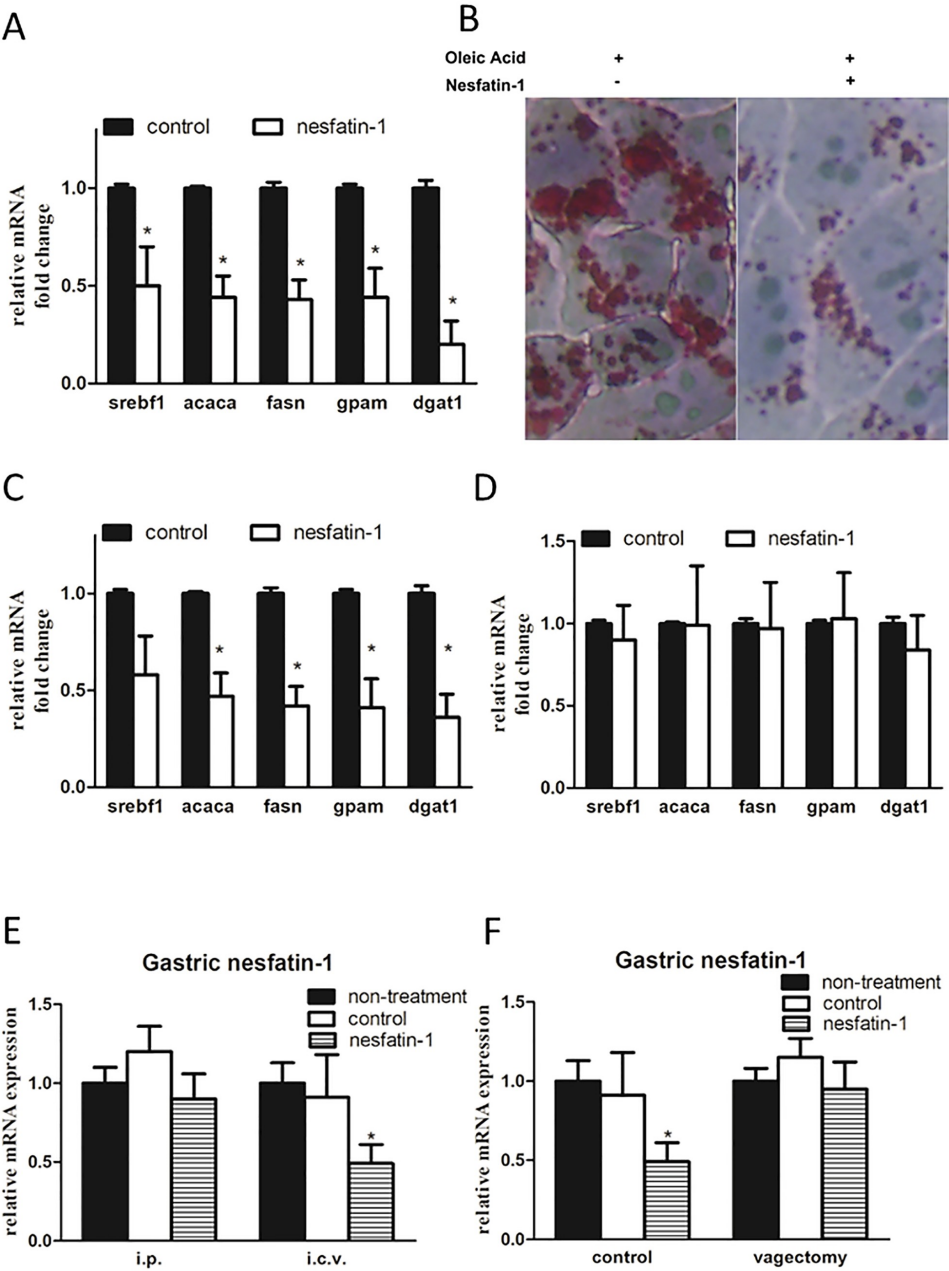
Effects of nesfatin-1 on liver lipid metabolism. A. *In vitro* stimulation of nesfatin-1 on hepatocytes. For each target gene, relative to control and normalized to GADPH. (Student t test, control vs nesfatin-1, n = 10 in each group, *p = 0.0038, Sref1; *p = 0.005, acaca; *p = 0.022, fasn; *p = 0.014 gpam; *p<0.001, dgat1). B. Oil red stain results of hepatocytes stimulated by Nesfatin-1. C. Hepatic PCR results of intraperitoneal injection of nesfatin-1. For each target gene, relative to control and normalized to GADPH. (Student t test, control vs nesfatin-1, n = 10 in each group, *p = 0.007, screbf; *p = 0.008, acaca; *p = 0.006, fasn; *p = 0.032, gpam, *p = 0.026, dgat1). D. Results of hepatic PCR after ICV of nesfatin-1. For each target gene, relative to control and normalized to GADPH. (Student t test, p>0.05 in all groups). E. Expression of nesfatin-1 in gastric tissue after intraperitoneal injection and ICV of nesfatin-1. For each group, relative to “non-treatment group” and normalized to GADPH, the i.p. group and i.c.v group were calculated and compared separately. (one-way ANOVA, *p<0.001, in ICV, n = 10 in each group). F. After vagectomy, the inhibitory effect of intraventricular injection of nesfatin-1 on the expression of nesfatin-1 in gastric tissue disappeared. For each group, relative to “non-treatment group” and normalized to GADPH, the i.p. group and i.c.v group were calculated and compared separately. (one-way ANOVA, *p<0.001, in Control, n = 10 in each group).

Cell experiments also showed that the treatment of nesfatin-1 significantly decreased the expression of lipogenesis-related genes and, thus, functions positively for NAFLD **(Fig 4A)**. Stimulation of nesfatin-1 in hepatocytes resulted in decreased lipid droplet size in the cells **(Fig 4B)**. To compare the different roles of the CNS and peripheral nesfatin-1 in the regulation of NAFLD, ICV and IP injection infusion effects were compared. The results suggested that infusion of nesfatin-1 through IP but not through ICV could improve liver lipid metabolism **(Fig 4C and 4D)**. This data suggested that peripheral nesfatin-1 plays the key role in the regulation of NAFLD. Further, infusion of nesfatin-1 via ICV but not IP could inhibit the expression of gastric nesfatin-1 **(Fig 4E)**. This results showed that although CNS nesfatin-1 did not directly affect NAFLD, it could regulate the expression of peripheral nesfatin-1, i.e. gastric nesfatin-1. However, this central-peripheral restriction effect is eliminated after vagectomy **(Fig 4F).**

## Discussion

Our previous study and those of other groups showed that nesfatin-1 could improve the metabolic state of the body [18,38]. But in patients/rats with obesity and diabetes, the expression of gastric nesfatin-1 is impaired [39–41]. According to our previous study [18], attenuated nesfatin-1 could be restored after RYGB. In this study, we further showed that this enhancement may be due to postprandial changes (Fig 1D and 1E). Moreover, this increase occurs immediately after surgery, suggesting surgery but not weight-loss effects are involved.

After RYGB, food bypass occurs, and there is induction of nesfatin-1 in the stomach after surgery. In our study, we found an increased postprandial nesfatin-1 level in the portal vein in rats that received RYGB surgery. Interestingly, there was no such increase in control rats, suggesting a novel role of surgery independent of weight loss. We attempted to determine whether the stomach bypass is related to the varied regulation pattern. Thus the modified RYGB was performed. The results suggested that cutting of the gastric vagus, but not food bypass, plays a key role in this nesfatin-1 regulation. Interestingly, vagectomy alone could show a similar pattern. Because nesfatin-1 is related to glucose and lipid metabolism, and our findings suggested that vagectomy alone could affect gastric nesfatin-1, we wondered whether vagectomy could function as a bariatric surgery. But the results seemed to be negative.

Previous studies suggested that the brain and gastro-duodenum are the main sources of nesfatin-1 and that it could restrict food intake [15–19]. However the mechanism of crosstalk of peripheral and central nesfatin-1 is largely unknown. Most of these studies focused on the CNS. However, because nesfatin-1 is also abundant in the digestive system [15], it could also play a role there. Interestingly, other studies showed that gastric nesfatin-1 expression is down-regulated after 24 hours of fasting in rodent models [42]. In addition, infusion of nesfatin-1 through the peripheral system could also inhibit food intake. Thus, the gastric peptide could also play a regulatory anorexigenic role as well. However, on the other hand, our study suggested that after food intake, gastric nesfatin-1 is slightly increased, and this increase seems to be rapidly controlled and limited in 2 to 3 hours. In contrast to this limited increase, RYGB rats showed a consistent induction of gastric nesfatin-1. Thus, RYGB changed the regulation pattern of gastric nesfatin-1 during food intake.

Furthermore, in our study, we found that infusion of nesfatin-1 through ICV could inhibit nesfatin-1 mRNA expression in the stomach, suggesting a negative control role of the CNS on the peripheral tissues. Our previous studies already proved that central nesfatin-1 could inhibit gastric acid secretion through the vagal mechanism system [18]. Thus it seems that the CNS affects the stomach through the vagus nerve. However, during RYGB, the vagus nerve is cut off. Thus, the direct anatomy link of the brain and stomach is severed. This may be the reason for the different regulation patterns of nesfatin-1 after surgery. To confirm this hypothesis, vagectomy was performed on rats. To our surprise, vagectomy alone could cause a similar gastric nesfatin-1 increase after food intake. In contrast to this, a modified RYGB surgery, in which the vagus nerve is preserved, showed unchanged nesfatin-1 expression, similar to that in the control groups. In light of the phenotypes of different surgery treatments, it could be concluded that the vagus nerve plays a vital role in the regulation of gastric nesfatin-1 secretion. It seems that during food intake, expression of nesfatin-1 in the CNS is induced, and in turn, it could inhibit similar changes in the stomach through the vagus nerve.

Nesfatin-1 and its precursor NUCB2 were first reported to induce dose-dependent anorexigenic effects [15–17]. In addition to the effects on stomach secretion and motility, studies have suggested that nesfatin-1 also directly affects glucose and lipid metabolism [18,19]. These metabolic effects are mainly related to peripheral infusion and both *in vivo* and *in vitro* experiments confirmed the anti-lipid accumulation role of nesfatin-1 on liver and hepatocytes. In our study, we observed increased postprandial nesfatin-1 after surgery. There was also a reduction of hepatic lipid accumulation *in vivo* as early as 3 days after surgery. Thus, there may be a correlation between the postprandial nesfatin-1 levels and NAFLD.

Many studies have confirmed that bariatric surgery results in weight loss and improvement of liver function tests in the long term [43,44]. However, in terms of NAFLD, reports suggested that it was better in RYGB than in adjustable gastric banding (AGB). The superiority of RYGB was primarily but not entirely explained by weight loss [45]. In line with these previous reports, our study showed that an increase in postprandial nesfatin-1 occurs immediately after surgery. When there are still no differences among treatment groups, the livers in the RYGB group already received the self-increased nesfatin-1 treatment, which, in turn has been confirmed to play positive roles in hepatic glucose and lipid metabolic.

Despite the fact that rapid improvement of lipid metabolism occurs after RYGB, the relief of NAFLD still largely relies on the general condition of the patient in the long term (i.e., body weight, blood glucose, and lipid). Thus, although vagectomy alone without gastric bypass could achieve a better state of liver lipid metabolism soon after surgery, we do not consider this surgery alone as a therapy for NAFLD. Body weight control is still the key in the long run. This finding is, however, still interesting because it indicated a very efficient mechanism by which surgery affects liver function that which could not be achieved by simple caloric limitation.

Collectively, our data show that that denervated vagus after RYGB could lead to higher gastric nesfatin-1 secretion after food intake, and this change resulted in improvement of NAFLD. This effect is independent of the weight-loss. Thus our study emphasizes the importance of the vagus nerve disconnection during surgery. Our observations again highlight the importance of the brain-gut-axis in surgery. However, the detailed mechanism of the crosstalk between the brain and gut through the vagus nerve in different metabolic surgery types is largely unknown and requires further discussion.

## Conclusion

Our study indicates that the gastric vagus nerve plays important roles during the relief of NAFLD after surgery. Food intake could stimulate secretion of both gastric and CNS nesfatin-1. CNS nesfatin-1 could inhibit gastric nesfatin-1 through the gastric vagus nerve. During RYGB, the vagus nerve is cut off, and the CNS-gut nesfatin-1 regulation pattern is affected due to vagectomy. Increased gastric nesfatin-1 could accumulate in the liver through the portal vein and promote lipid metabolism in the liver. Finally, this changed pattern could relieve NAFLD rapidly after surgery. This finding may be an indication that the gastric vagus nerve may be a key for gut hormone regulation, and vagectomy could be a vital surgery step for the rapid effects of relief from NAFLD.

## Supporting information

S1 Fig A-D. Schematic diagram of different surgical models.
(TIF)

S2 Fig Tree diagram of the details of the groups and subgroups. (TIF)

S3 Fig Tree diagram of the details of the groups and subgroups. (TIF)

S4 Fig Tree diagram of the details of the groups and subgroups. (TIF)

S5 Fig A. control group validation: Results of hepatic PCR of normal rats after been provided a gavage of glucose or water (Student t test, p>0.05 in all groups). B. Comparison of liver sections between the chow group and the HFD group.
(TIF)

S6 Fig Original uncropped and unadjusted western-blot images. (TIF)

S1 File (DOCX)

S2 File (DOCX)
