# Supplementary figures and images for: RYGB increases postprandial gastric nesfatin-1 and rapid relieves NAFLD via gastric nerve detachment
Source: PLoS One. 2020 Dec 10;15(12):e0243640. doi: 10.1371/journal.pone.0243640 (PMC7728189; doi:10.1371/journal.pone.0243640)

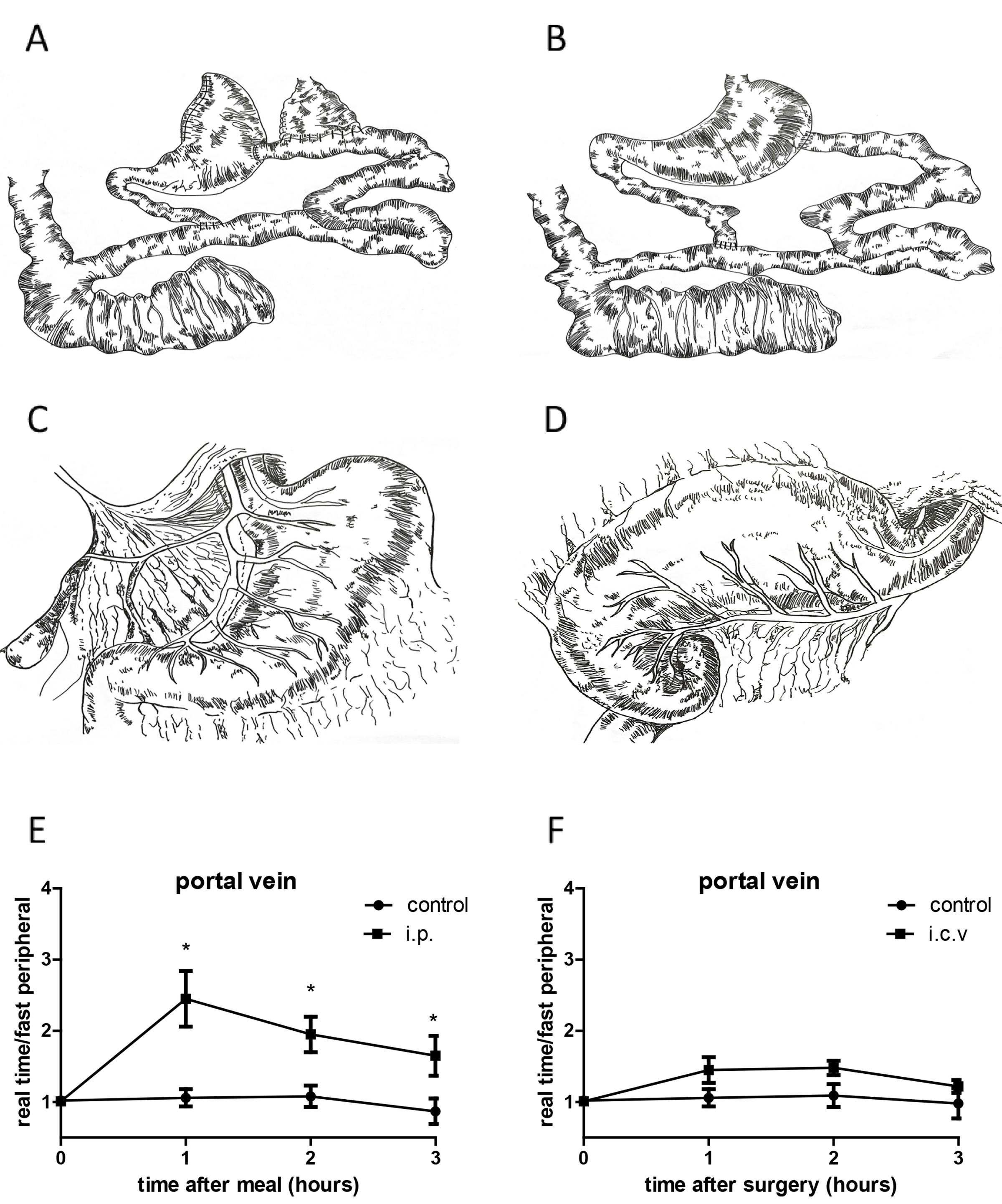

Supplement: S1 Fig — A-D. Schematic diagram of different surgical models. (TIF) [file pone.0243640.s001.tif]

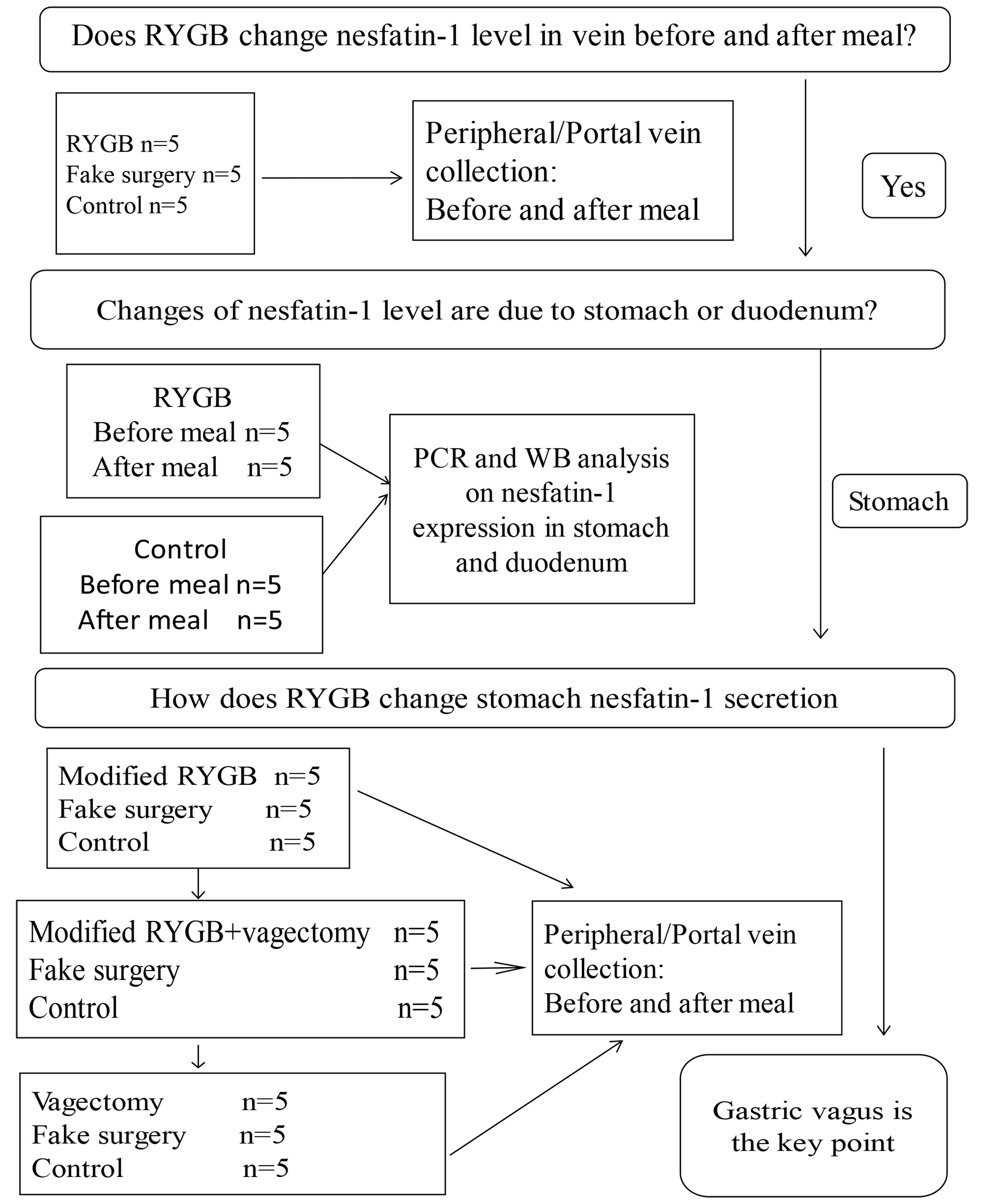

Supplement: S2 Fig — (TIF) [file pone.0243640.s002.tif]

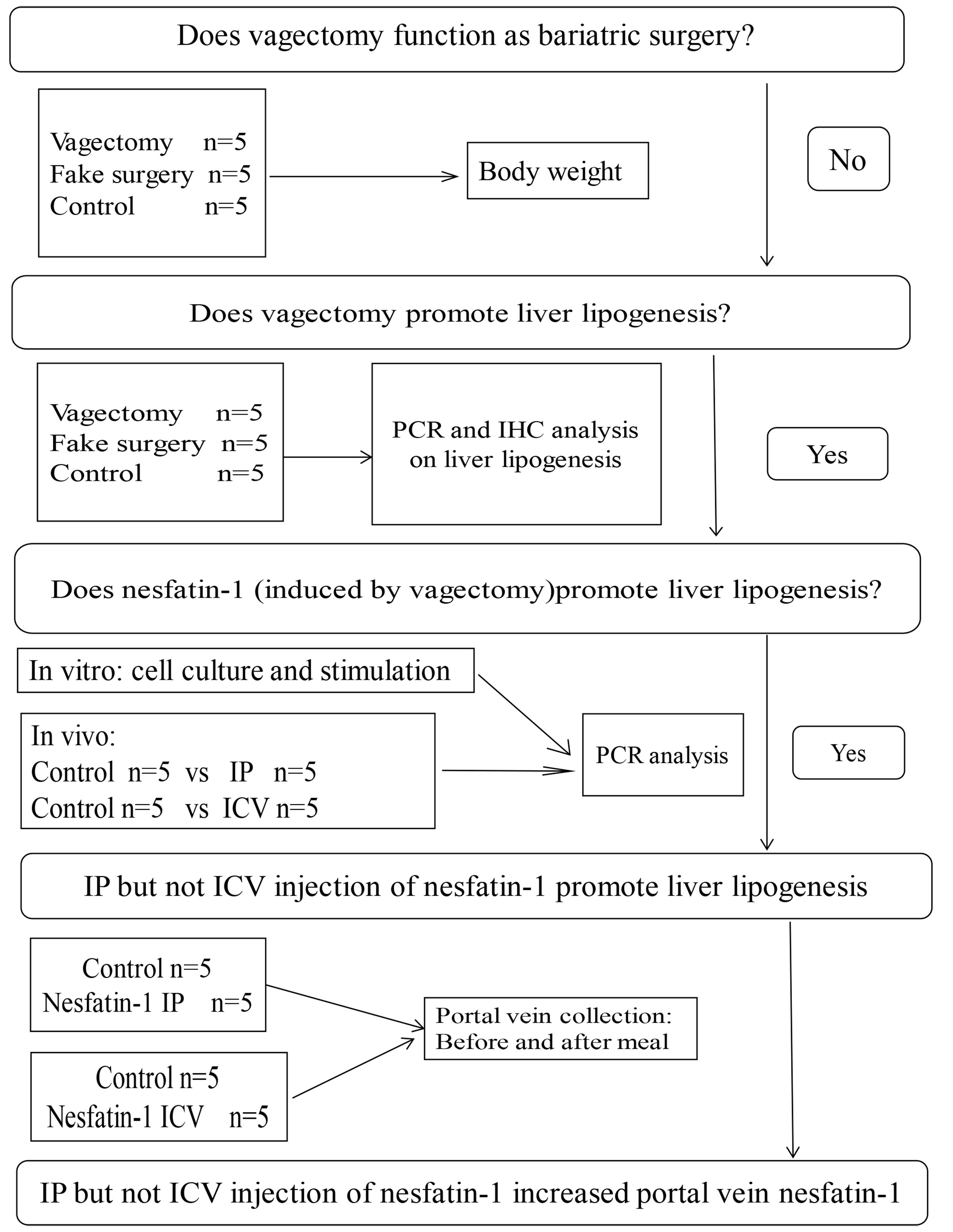

Supplement: S3 Fig — (TIF) [file pone.0243640.s003.tif]

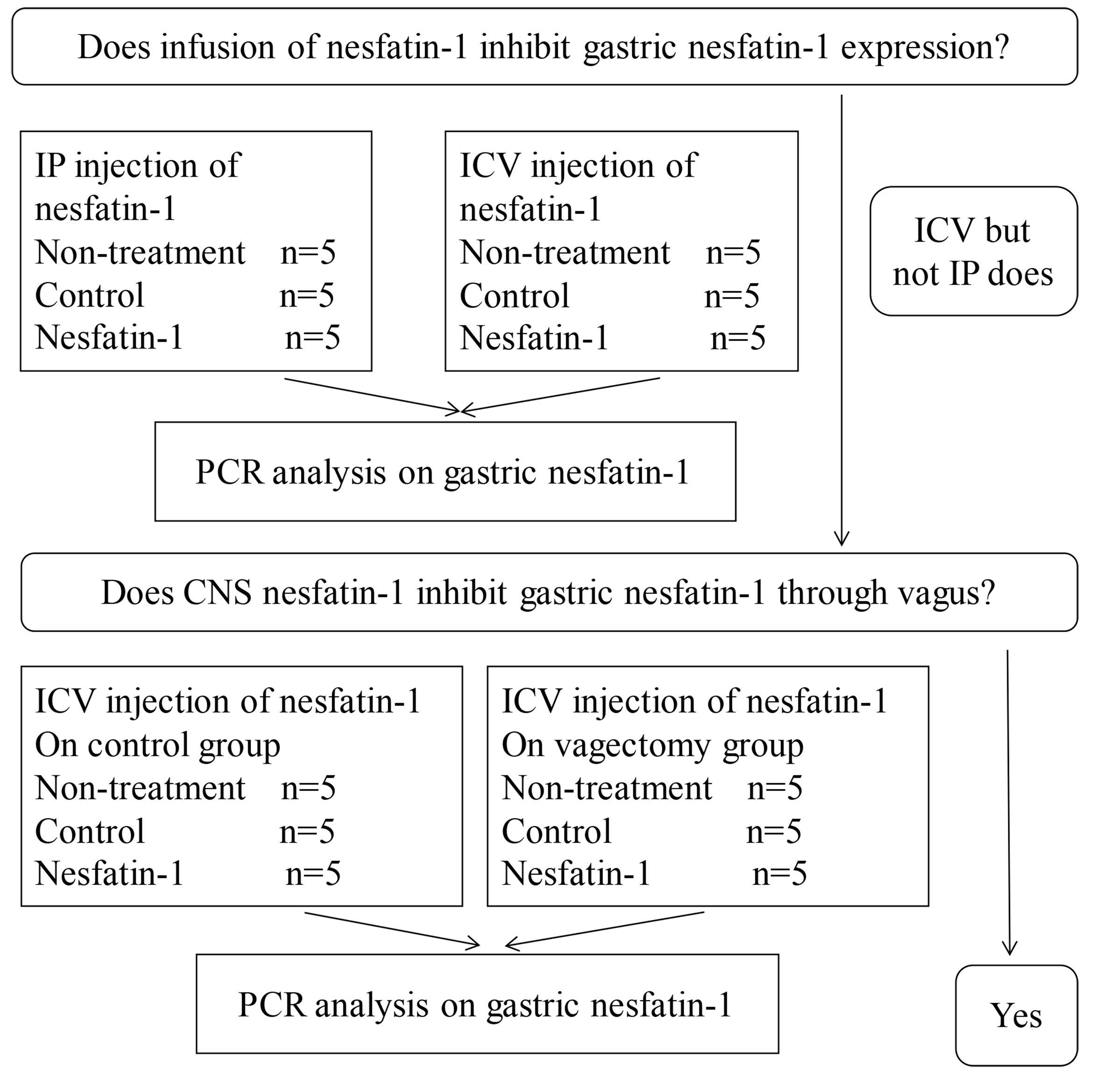

Supplement: S4 Fig — (TIF) [file pone.0243640.s004.tif]

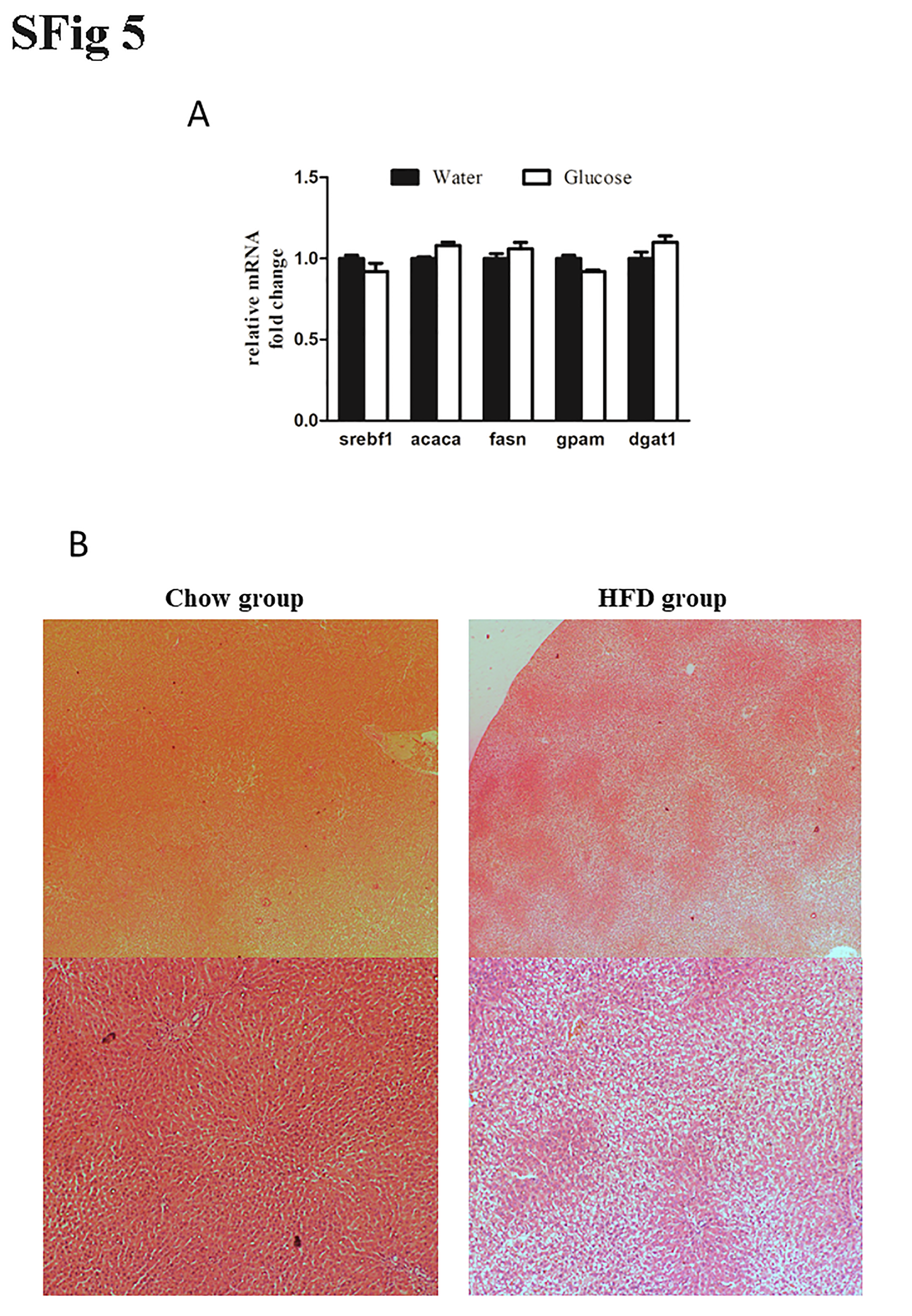

Supplement: S5 Fig — A. control group validation: Results of hepatic PCR of normal rats after been provided a gavage of glucose or water (Student t test, p>0.05 in all groups). B. Comparison of liver sections between the chow group and the HFD group. (TIF) [file pone.0243640.s005.tif]

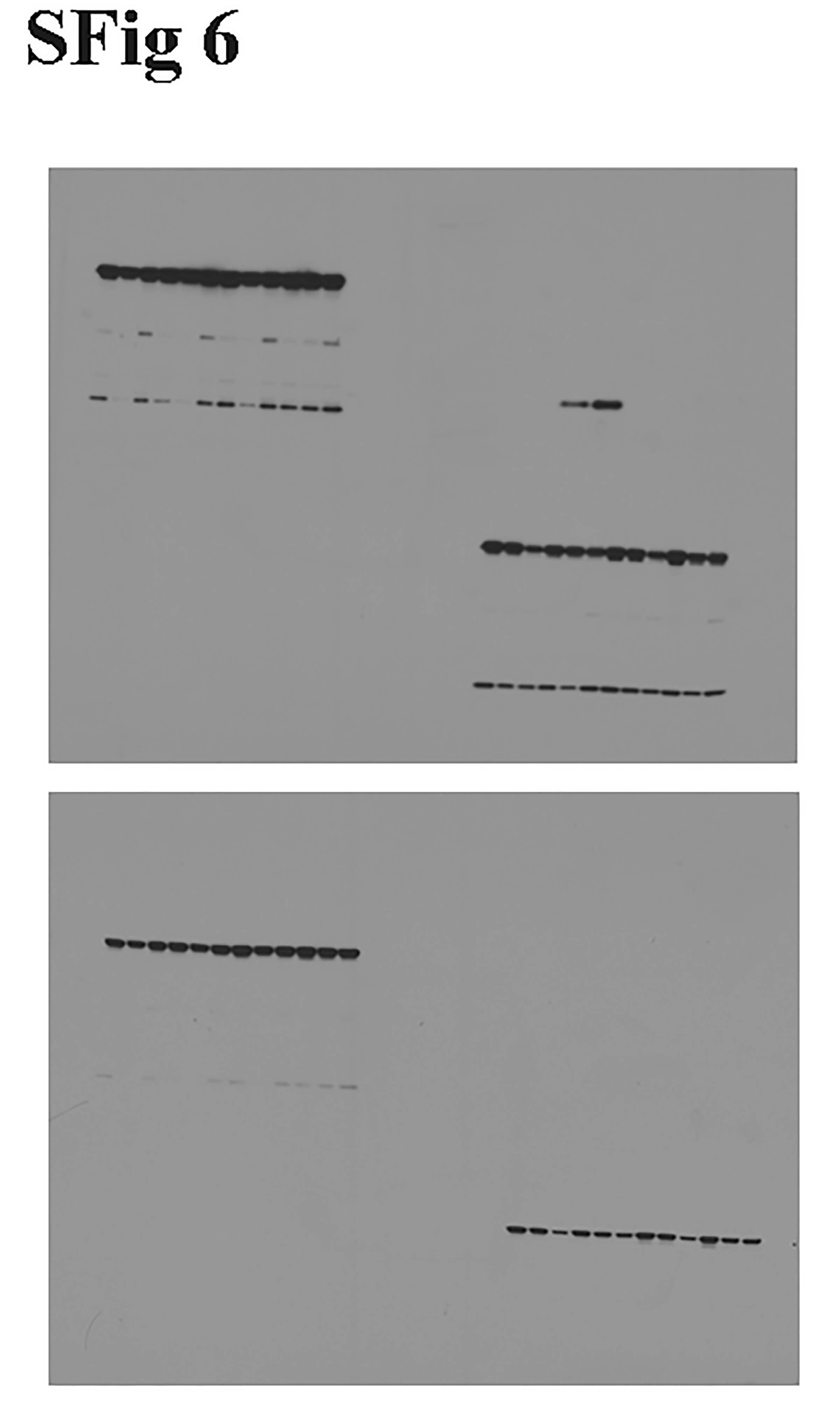

Supplement: S6 Fig — (TIF) [file pone.0243640.s006.tif]
