# Supplementary material for: RYGB increases postprandial gastric nesfatin-1 and rapid relieves NAFLD via gastric nerve detachment
Source: PLoS One. 2020 Dec 10;15(12):e0243640. doi: 10.1371/journal.pone.0243640 (PMC7728189; doi:10.1371/journal.pone.0243640)
Supplement: S1 File — (DOCX) [file pone.0243640.s007.docx]

Fig1A one-way ANOVA group df=2 total df=30 error df=27

| Time point | F | p |
| --- | --- | --- |
| 0 | 0.343 | 0.713 |
| 0.5 | 2.7896 | 0.079 |
| 1 | 2.727 | 0.083 |
| 1.5 | 1.051 | 0.363 |
| 2 | 0.261 | 0.772 |
| 2.5 | 0.536 | 0.591 |
| 3 | 1.271 | 0.297 |

Fig1B one-way ANOVA group df=2 total df=30 error df=27

| Time point | F | p |
| --- | --- | --- |
| 0 | 2.675 | 0.087 |
| 0.5 | 3.912 | 0.065 |
| 1 | 7.100 | 0.003 |
| 1.5 | 7.16 | 0.003 |
| 2 | 18.25 | <0.001 |
| 2.5 | 31.694 | <0.001 |
| 3 | 68.157 | <0.001 |

Fig1C Student t test df=18

| subject | t | p |
| --- | --- | --- |
| stomach | 0.257 | 0.800 |
| duodenum | -1.178 | 0.103 |

Fig1D Student t test df=18

| subject | t | p |
| --- | --- | --- |
| stomach | -8.292 | <0.001 |
| duodenum | -0.445 | 0.662 |

Fig2B one-way ANOVA group df=2 total df=30 error df=27

| Time point | F | p |
| --- | --- | --- |
| 0 | 1.019 | 0.375 |
| 0.5 | 1.903 | 0.169 |
| 1 | 2.175 | 0.133 |
| 1.5 | 0.620 | 0.545 |
| 2 | 0.684 | 0.513 |
| 2.5 | 2.763 | 0.082 |
| 3 | 1.545 | 0.232 |

Fig2C one-way ANOVA group df=2 total df=30 error df=27

| Time point | F | p |
| --- | --- | --- |
| 0 | 1.306 | 0.287 |
| 0.5 | 4.495 | 0.021 |
| 1 | 4.187 | 0.026 |
| 1.5 | 19.892 | <0.001 |
| 2 | 53.58 | <0.001 |
| 2.5 | 37.853 | <0.001 |
| 3 | 11.394 | <0.001 |

Fig2D one-way ANOVA group df=2 total df=30 error df=27

| Time point | F | p |
| --- | --- | --- |
| 0 | 0.911 | 0.414 |
| 0.5 | 4.342 | 0.023 |
| 1 | 0.957 | 0.397 |
| 1.5 | 8.106 | 0.02 |
| 2 | 27.756 | <0.001 |
| 2.5 | 24.673 | <0.001 |
| 3 | 18.389 | <0.001 |

Fig3A repeated ANOVA n=6 in each group, 3 groups

For “time after surgery”: F=70.006 P<0.001: a significantly and consistent increase after surgery.

For differences among 3 groups: F=0.013 p=0.987: no differences among 3 groups

Fig3B one way ANOVA group df=2 total df=18 error df=15

| subject | F | p |
| --- | --- | --- |
| Screbf1 | 25.062 | <0.001 |
| Acaca | 62.129 | <0.001 |
| Fasn | 69.886 | <0.001 |
| Gpam | 32.167 | <0.001 |
| Dgat1 | 116.003 | <0.001 |

Fig4 A Student t test df=8

| subject | t | p |
| --- | --- | --- |
| Screbf1 | 2.475 | 0.038 |
| Acaca | 3.84 | 0.005 |
| Fasn | 2.934 | 0.022 |
| Gpam | 3.15 | 0.014 |
| Dgat1 | 5.52 | <0.001 |

Fig4 C Student t test df=8

| subject | t | p |
| --- | --- | --- |
| Screbf1 | 3.626 | 0.007 |
| Acaca | 3.544 | 0.008 |
| Fasn | 3.746 | 0.006 |
| Gpam | 2.665 | 0.032 |
| Dgat1 | 4.097 | 0.026 |

Fig4 D Student t test df=8

| subject | t | p |
| --- | --- | --- |
| Screbf1 | 1.774 | 0.150 |
| Acaca | -0.077 | 0.941 |
| Fasn | -1.481 | 0.177 |
| Gpam | 0.043 | 0.967 |
| Dgat1 | 1.043 | 0.328 |

Fig4E one-way ANOVA group df=2 total df=18 error df=17

| subject | F | p |
| --- | --- | --- |
| IP | 1.117 | 0.326 |
| ICV | 15.712 | <0.001 |

Fig4F one-way ANOVA group df=2 total df=18 error df=17

| subject | F | p |
| --- | --- | --- |
| Control | 32.288 | <0.001 |
| Vagectomy | 0.466 | 0.632 |
