# Supplementary material for: RYGB increases postprandial gastric nesfatin-1 and rapid relieves NAFLD via gastric nerve detachment
Source: PLoS One. 2020 Dec 10;15(12):e0243640. doi: 10.1371/journal.pone.0243640 (PMC7728189; doi:10.1371/journal.pone.0243640)
Supplement: S2 File — (DOCX) [file pone.0243640.s008.docx]

**Introduction for the uncropped WB images**

Uncropped WB image1: stomach tissue

Left line3: nesfatin-1 Right line2: GADPH

| RYGB  After meal | RYGB before meal | RYGB  After meal | RYGB before meal | RYGB  After meal | RYGB before meal | RYGB  After meal | RYGB before meal | Control after meal | Control before meal | Control after meal | Control before meal |
| --- | --- | --- | --- | --- | --- | --- | --- | --- | --- | --- | --- |

Uncropped WB image2: duodenum tissue

Left line: GADPH Right line: nesfatin-1

| RYGB  After meal | RYGB before meal | RYGB  After meal | RYGB before meal | RYGB  After meal | RYGB before meal | RYGB  After meal | RYGB before meal | Control after meal | Control before meal | Control after meal | Control before meal |
| --- | --- | --- | --- | --- | --- | --- | --- | --- | --- | --- | --- |
